# Mitochondrial quality control, promoted by PGC-1*α*, is dysregulated by Western diet-induced obesity and partially restored by moderate physical activity in mice

**DOI:** 10.14814/phy2.12470

**Published:** 2015-07-15

**Authors:** Nicholas P Greene, David E Lee, Jacob L Brown, Megan E Rosa, Lemuel A Brown, Richard A Perry, Jordyn N Henry, Tyrone A Washington

**Affiliations:** 1 Integrative Muscle Metabolism Laboratory, Human Performance Laboratory, Department of Health, Human Performance and Recreation, University of ArkansasFayetteville, Arkansas; 2 Exercise Muscle Biology Laboratory, Human Performance Laboratory, Department of Health, Human Performance and Recreation, University of ArkansasFayetteville, Arkansas

**Keywords:** Autophagy, dynamics, insulin resistance, mitochondrial biogenesis

## Abstract

Skeletal muscle mitochondrial degeneration is a hallmark of insulin resistance/obesity marked by lost function, enhanced ROS emission, and altered morphology which may be ameliorated by physical activity (PA). However, no prior report has examined mitochondrial quality control regulation throughout biogenesis, fusion/fission dynamics, autophagy, and mitochondrial permeability transition pore (MPTP) in obesity. Therefore, we determined how each process is impacted by Western diet (WD)-induced obesity and whether voluntary PA may alleviate derangements in mitochondrial quality control mechanisms. Despite greater mitochondrial content following WD (COX-IV and Cytochrome C), induction of biogenesis controllers appears impaired (failed induction of PGC-1*α*). Mitochondrial fusion seems diminished (reduced MFN2, Opa1 proteins), with no significant changes in fission, suggesting a shift in balance of dynamics regulation favoring fission. Autophagy flux was promoted in WD (reduced p62, increased LC3II:I ratio); however, mitophagy marker BNIP3 is reduced in WD which may indicate reduced mitophagy despite enhanced total autophagy flux. MPTP regulator *Ant* mRNA is reduced by WD. Few processes were impacted by physical activity. Finally, mitochondrial quality control processes are partially promoted by PGC-1*α*, as PGC-1*α* transgenic mice display elevated mitochondrial biogenesis and autophagy flux. Additionally, these mice exhibit elevated *Mfn1* and *Opa1* mRNA, with no change in protein content suggesting these factors are transcriptionally promoted by PGC-1*α* overexpression. These data demonstrate dysfunctions across mitochondrial quality control in obesity and that PGC-1*α* is sufficient to promote multiple, but not necessarily all, aspects of mitochondrial quality control. Mitochondrial quality control may therefore be an opportune target to therapeutically treat metabolic disease.

## Introduction

Obesity and type 2 diabetes mellitus (T2DM) are at pandemic levels in Western societies (Centers for Disease Control and Prevention, 2013) and lead to increased risk for cardiovascular disease and premature death along with poor quality of life (American Diabetes Association, 2014a,b). Skeletal muscle is a major metabolic tissue, comprising 40–60% of body weight, particularly susceptible to metabolic dysfunctions and may be the sight of onset in insulin resistance (DeFronzo and Tripathy 2009). In muscle, obesity/T2DM are associated with reduced glucose uptake, abnormal protein turnover, dysregulated lipid metabolism, and mitochondrial dysfunction (Koves et al. 2008; Anderson et al. 2009; Nilsson et al. 2010, 2013; Greene et al. 2014). Skeletal muscle mitochondria exhibit altered morphology (Kelley et al. 2002), impaired mitochondrial quality (health and functionability of the network) (Laker et al. 2014) and function (Kelley et al. 2002; Yokota et al. 2009), and enhanced reactive oxygen species (ROS) and lipid byproduct emission (Koves et al. 2008; Anderson et al. 2009) in obesity/T2DM. We reported mitochondrial anabolic resistance to exercise in obese Zucker rats despite induction of peroxisome proliferator-activated receptor *γ* coactivator-1*α* (PGC-1*α*), the master regulator of mitochondrial biogenesis, while also displaying elevated mitochondrial content (Nilsson et al. 2010; Greene et al. 2014). As such, identification of dysregulated points in maintenance of mitochondrial quality is critical to preserve cellular health. Mitochondrial quality is mediated by four major processes: biogenesis, dynamics, autophagy, and the mitochondrial permeability transition pore (MPTP) (Yan et al. 2012). However, to our knowledge, no single study has reported the impact of diet-induced obesity/T2DM, or physical activity (PA), on the total mitochondrial quality control processes.

Mitochondrial biogenesis has been the focus of significant efforts, culminating in the discovery of PGC-1*α* (Puigserver et al. 1998) and the realization that exercise-induced mitochondrial biogenesis functions through PGC-1*α* (Lin et al. 2002; Geng et al. 2010). However, mitochondria function optimally in a combined network where they are able to share components (Yan et al. 2012). Therefore, coordinated processes to regulate the maintenance of the network are imperative for mitochondrial quality. This regulation occurs through mitochondrial dynamics, autophagy, and MPTP. Mitochondrial dynamics is the process of adding and removing mitochondrial components to and from the network by fusion and fission. Fusion is regulated by the mitofusins -1 and -2 (MFNs 1 and 2) and optic atrophy 1 (Opa1) (Westermann 2010). For fission to occur mitochondrial fission factor (MFF) and Fission1 (Fis1) promote recruitment of Drp1 to the mitochondria to perform fission (Westermann 2010).

Autophagy is a highly conserved process for degradation of organelles and macromolecules (Klionsky 2005). The primary forms of autophagy include nonselective autophagy induced by starvation; and, selective autophagy removing damaged organelles, such as mitochondria, and aggregate proteins (Mizushima et al. 2008). Autophagy is regulated and performed by autophagy-related genes (Atgs), including Atg6 (hereafter referred to as Beclin, mammalian name). Within the mitochondria, the BCL2/Adenovirus E1B 19 KDa Protein-Interacting Protein 3 (BNIP3) specifically regulates mitochondrial autophagy (mitophagy) (Romanello et al. 2010). Autophagy flux is commonly estimated by the shift in LC3 between lipidated (LC3-II) and delipidated forms (LC3-I), whereby an increase in LC3II:I ratio represents enhanced flux and reduced content of the cargo protein p62/SQSTM1 which itself is degraded upon carrying tagged cellular components to the autophagosome (Mizushima et al. 2008).

Mitochondrial permeability transition pore is a transient opening allowing the passive diffusion of molecules <1.5 kDa in and out of the mitochondria. Physiologically, the MPTP likely serves as a pressure release valve allowing mitochondria to release built up ROS, lipid byproducts, and other unnecessary materials. Under pathophysiologic conditions, the MPTP probably serves a self-destruct mechanism resulting in energetic dysfunction, swelling, and rupture (Kwong and Molkentin 2015). Canonically, the MPTP is made up of the Adenine Nucleotide Transporter (ANT), the Voltage-Dependent Anion Channel (VDAC), and Cyclophilin D (CypD) (Elrod and Molkentin 2013), though other components have been identified (Alavian et al. 2014). Notably, MPTP inhibition protects against diet-induced insulin resistance (Taddeo et al. 2014). However, the precise role of MPTP in metabolic disease is not yet completely understood.

While obesity and T2DM are tied to poor mitochondrial quality, PA is associated with improved mitochondrial quality. Laker et al. (Laker et al. 2014) used reporter genes to visualize mitochondrial deterioration following high-fat feeding which could be ameliorated by exercise training. Other prior reports suggest PA promotes the expression of regulators of biogenesis (Akimoto et al. 2005; Pogozelski et al. 2009; Greene et al. 2012, 2014), fusion (Ding et al. 2010; Perry et al. 2010), fission (Perry et al. 2010), and autophagy (He et al. 2012; Lira et al. 2013). However, whether obesity/T2DM impairs mitochondrial quality control mechanisms and if such impairment may be alleviated by PA is as of yet untested. Additionally, *in vitro* data suggest that PGC-1*α* may transcriptionally regulate MFN1 and MFN2 (Cartoni et al. 2005), while data from PGC-1*α* overexpressing mice suggests PGC-1*α* promotes basal autophagy and mitophagy (Lira et al. 2013). Therefore, PGC-1*α* may function in all aspects of mitochondrial quality control. However, to our knowledge, no single study has been conducted to examine if PGC-1*α* is sufficient to promote total mitochondrial quality control processes.

Therefore, our purpose was to examine the impact of diet-induced obesity/T2DM and PA on the regulatory network for mitochondrial quality control. We hypothesized diet-induced obesity would impair regulation of each process directing mitochondrial quality control and this impairment would be partially ameliorated by PA. Finally, we assessed if PGC-1*α* is indeed sufficient to promote total mitochondrial quality control processes in basal conditions rather than just biogenesis. These studies provide novel evidences for disruptions in the regulation of mitochondrial quality control by obesity and mechanisms controlling total mitochondrial quality.

## Materials and Methods

### Animals and interventions

All methods were approved by the Institutional Animal Care and Use Committee of the University of Arkansas.

#### Western diet and voluntary wheel running

C57BL6/J male mice (*n* = 40) were purchased (Jackson Laboratories, Bar Harbor, ME) at 7 weeks of age. Animals were housed in a secure, temperature- and humidity-controlled environment and maintained on a 12:12 h light–dark cycle with food and water provided ad libitum. To induce obesity and insulin resistance, at 8 weeks of age animals were divided between normal laboratory chow (NC, Teklad 22/5 Rodent Diet, 8640, Teklad Diets, Madison, WI) or Western diet (WD, 42% kcal by fat plus 1.5 g cholesterol/kg diet, D12079B, Research Diets, New Brunswick, NJ) similar to previous designs (Song et al. 2010; Funai et al. 2013; Stanford et al. 2013). Animals were allowed ad libitum access to food throughout and feed was weighed weekly to monitor intake. After 4 weeks of diet, animals from both feed groups were divided between sedentary (SED) and PA (“exercise”) conditions. Lifestyle PA was performed by 4 weeks of voluntary wheel running (VWR) using a home cage wheel running system with Hall Effect Sensors connected to an eight channel wheel counterinterface and MDI Multi Interface Software (Columbus Instruments, Columbus, OH). Throughout VWR all animals were singly housed. This design allowed the induction of obesity/insulin resistance prior to attempts to ameliorate this condition with lifestyle activity and created four experimental groups: NC-SED, NC-VWR, WD-SED, WD-VWR; *n* = 10/group.

#### PGC-1*α* muscle-specific overexpressing mice

A breeding pair of mice with muscle-specific overexpression of PGC-1*α* under the muscle creatine kinase promoter (MCK-PGC-1*α*) was a generous gift from Dr. Bruce Spiegelman (Dana-Farber Cancer Institute, Boston, MA). Mice were bred in the PI’s laboratory, weaned at 3 weeks age and genomic DNA was isolated from the tail followed by PCR-based genotyping designed for the exogenous MCK-PGC-1*α* construct using the forward primer 5′-GCAGGATCACATAGGCAGGATGTGGCC-3′ and reverse primer 5′-GGAAGATCTGGGCAAAGAGGCTGGTCC-3′ as described by Lira et al. 2013). Male MCK-PGC-1*α* mice (*n* = 9) and wild type (WT, *n* = 12) littermates were raised to 12 weeks of age on standard laboratory chow and muscle samples harvested and processed for protein and RNA.

### Physiological testing

#### Glucose tolerance test

Intraperitoneal (I.P.) glucose tolerance tests (GTT) were performed at 15 weeks of age in all mice corresponding to 8 weeks diet/4 weeks exercise, respectively. Prior to GTTs mice were fasted 6 h and then given a bolus of glucose (1.5 g glucose/kg body weight dissolved in sterile saline) by I.P. injection. Blood samples were collected from tail vein and assessed for glucose concentration before injection (0 min) and 30, 60, and 120 min post-injection using a Relion® Ultima diabetic glucometer (Abbott Diabetes Care, Alameda, CA). Data were analyzed as blood glucose area under the curve.

#### Fatiguing treadmill test

Graded exercise test (GXT) was a modified version of that previously reported by Lira et al. 2013. Briefly, animals were positioned into custom built lanes placed upon a standard motorized human treadmill. Animals were exercised on the treadmill for 10 min at 13.4 m/min for three consecutive days to familiarize with the exercise. On the fourth day, the treadmill test began with a 0% grade at 13.4 m/min; after 10 min speed was increased to 18.8 m/min and grade to 5% at which point grade was maintained at 5% for the remainder of the test. Speed was increased by 2.7 m/min every 30 min until reaching 26.8 m/min, speed and grade then remained constant until volitional exhaustion. A brush located at the end of each treadmill lane was used to encourage animals to run and the test was terminated when the mouse stopped responding to tail brushing continuously for 20 sec.

### Tissue collection

At the appropriate age (16 weeks in WD/VWR animals following total 8 weeks intervention or 12 weeks in MCK-PGC-1*α* animals) animals were humanely euthanized. Running wheels were removed from cages 24 h prior to tissue collection. Hindlimb muscles including gastrocnemius, tibialis anterior, soleus, plantaris, extensor digitorum longus (EDL), and epididymal fat samples were quickly collected, weighed, and snap frozen in liquid nitrogen. Animals’ tibias were then collected and measured for total length as a common surrogate for body size and tissue weights normalized to the average length of both tibias (Yin et al. 1982; Reiss et al. 1996; Okutsu et al. 2014). Gastrocnemius muscle samples were pulverized in liquid nitrogen prior to preparation for RNA or protein analysis. Gastrocnemius muscle was selected as a commonly accepted mixed fiber-type muscle responsive to metabolic interventions such as diet and PA.

### RNA isolation, cDNA synthesis and real-time RT-PCR

RNA isolation, cDNA synthesis, and real-time RT-PCR were performed as previously reported (Washington et al. 2013; Greene et al. 2014) with minor modifications. Briefly, RNA was extracted from gastrocnemius muscle with Trizol reagent (Life Technologies, Grand Island, NY) as suggested by the manufacturer and isolated using Ambion Purelink RNA minikit (Life Technologies, Applied Biosystems, Grand Island, NY). Total RNA was DNase treated and concentration and purity were determined using a BioTek Take3 micro-volume microplate with a BioTek PowerWave XS microplate reader (BioTek Instruments Inc., Winooski, VT). cDNA was reverse transcribed from 1 *μ*g of total RNA using Quanta qScript cDNA Supermix (Quanta Biosciences, Gaithersburg, MD) according to the manufacturer instructions. Real-Time Polymerase Chain Reaction (PCR) was performed using the StepOne Real-Time PCR system (Life Technologies, Applied Biosystems, Grand Island, NY) and results were analyzed using stepone Software. cDNA was amplified in a 25 *μ*L reaction containing appropriate primer pairs or probes and SYBR Green or TaqMan Universal Mastermix as appropriate (Applied Biosystems). The samples were incubated at 95°C for 4 min, followed by 40 cycles of denaturation, annealing, and extension at 95, 60, and 72°C. Fluorescence was measured at the end of the extension step for each cycle. Fluorescence-labeled probes for PGC-1*α* (mm01208835_m1), COX-IV (mm01250094_m1), PPAR*δ* (mm00803184_m1), TFAM (Rn00580051_m1, rat probe confirmed by agarose gel for mouse) (FAM dyes); and HPRT (mm01545399_m1) VIC dye) were purchased from Applied Biosystems. SYBR Green primers were designed using Primer-BLAST through PubMed. Custom primers were designed to produce amplicons between 70 and 150 bp with melting temperatures at 60°C, forced to span exon–exon junctions and produced pairs with potential binding on unintended targets were eliminated from consideration. Amplicon products were separated on agarose gel and visualized using ethidium bromide under ultraviolet light to confirm the existence of a single band at the intended size; images were acquired using a customized gel documentation system with a Nikon Coolpix L820 digital camera. Custom primer pairs are shown in Table1. Cycle Threshold (C_t_) was determined and the ΔC_t_ value calculated as the difference between C_t_ value and HPRT C_t_ value. HPRT C_t_ was not different among experimental groups. Final quantification of gene expression was calculated using the ΔΔC_t_ method. Relative quantification was calculated as 2^−ΔΔCt^.

**Table 1:** Primer pairs used for SYBR-Green-based quantitative Real-Time RT-PCR

Target	Forward Primer	Reverse Primer	Tm (°C)	Amplicon length (bp)	RefSeq ID#
*Nrf2*	AGAGCAACTCCAGAAGGAACAG	TGTGGGCAACCTGGGAGTAG	60	146	NM_010902.3
*Pparα*	ATTTGCTGTGGAGATCGGC	GCTTTGGGAAGAGGAAGGTGT	60	130	NM_001113418.1
*Mfn1*	TTGCCACAAGCTGTGTTCGG	TCTAGGGACCTGAAAGATGGGC	60	148	NM_024200.4
*Mfn2*	AGAGGCAGTTTGAGGAGTGC	ATGATGAGACGAACGGCCTC	60	103	NM_001285920.1
*Opa1*	TCTGAGGCCCTTCTCTTGTT	TCTGACACCTTCCTGTAATGCT	60	98	NM_001199177.1
*Fis1*	ACGAAGCTGCAAGGAATTTTGA	AACCAGGCACCAGGCATATT	60	98	NM_001163243.1
*Mff*	TCGGGTCTGTCCTCCCCATA	CAACACAGGTCTGCGGTTTTCA	60	145	NM_029409.2
*Drp1*	TCACCCGGAGACCTCTCATT	TGCTTCAACTCCATTTTCTTCTCC	60	89	NM_001025947.2
*Beclin*	CTCCATTACTTACCACAGCCCA	AAATGGCTCCTCTCCTGAGT	60	77	NM_019584.3
*Atg7*	TGACCTTCGCGGACCTAAAG	AGGGCCTGGATCTGTTTTGG	60	139	NM_001253717.1
*Bnip3*	GAAGCGCACAGCTACTCTCA	TCCAATGTAGATCCCCAAGCC	60	142	NM_009760.4
*Vdac*	CCTCCCACATACGCCGATCT	TTAAGCCAAAGCCGTAGCCC	60	70	NM_011694.4
*CypD*	CTCCAACTCCAAGAACCCGC	TAAAACAATTCGGCCAACTCGC	60	73	NM_026352.3
*Ant*	GTAGGCAAGAGCAAACGAGC	AAGCTCAAAGCCTGATCCCC	60	78	NM_007450.4

### Isolation of protein and immunoblotting

Gastrocnemius muscles were homogenized with glass homogenizers in 0.2 ml of complete protein loading buffer containing 50 mM Tris·HCl, pH 6.8, 1% sodium dodecyl sulfate (SDS), 10% glycerol, 20 mM dithiothreitol, 127 mM 2-mercaptoethanol, and 0.01% bromophenol blue, supplemented with protease inhibitors (Roche, Nutley, NJ) and phosphatase inhibitors (Sigma-Aldrich, St. Louis, MO) per Alliance for Cellular Signaling protocols. The muscle homogenates were transferred to microfuge tubes, heated for 5 min at 95°C, and centrifuged in a microfuge for 5 min at 13 000 rpm at room temperature. Protein concentration of each sample was determined using the RC/DC assay (Bio-Rad, Hercules, CA), and 40 *μ*g protein was resolved on an 8–15% (depending on molecular weight of protein of interest) SDS-polyacrylamide gel (PAGE) and transferred to PVDF membrane (Thermo Scientific, Rockford, IL). Membranes were incubated in blocking solution [5% nonfat dried milk dissolved in Tris-buffered saline (TBS)]. The following antibodies were then diluted in blocking solution: COX-IV (Cell Signaling Technology-4844, Danvers, MA), PGC-1*α* (Santa Cruz Biotechnologies sc-13067, Santa Cruz, CA), PPAR*α* (Santa Cruz sc-9000), PPAR*β*/*δ* (Santa Cruz sc-7197), TFAM (Cell Signaling 7495), MFN1 (Santa Cruz sc-50330), MFN2 (Santa Cruz sc-50331), Fis1 (Santa Cruz sc-48865), Opa1 (Santa Cruz sc-367890), LC3a/b (Cell Signaling 4108), BNIP3 (Cell Signaling 3769), p62/SQSTM1 (Sigma Aldrich p0067, St. Louis, MO), Beclin (Cell Signaling 3738), Drp1 (Cell Signaling 14647), Cytochrome C (Cell Signaling 4272), GAPDH (Cell Signaling 2118). All immunoblots for PGC-1*α* were confirmed against a positive control of MCK-PGC-1*α* muscle sample. Similar to prior findings (Lira et al. 2013), we observed the presence of two bands for p62 and based on that prior report, we have analyzed the upper band as p62. BNIP3 has previously been seen to form a homodimer (Chen et al. 1997) with a monomer seen at 22–28 kDa and the homodimer at 50–56 kDa, therefore, BNIP3 content was taken as the sum density of both bands. The membranes were washed and incubated with appropriate secondary antibodies coupled to horseradish peroxidase (Li-Cor Biosciences, Lincoln, NE or Santa Cruz), developed using chemiluminescence and imaged using a FluorChem M Blot Imaging System (Protein Simple, San Jose, CA). Content of proteins was normalized to GAPDH and to a protein standard loaded on each gel (mouse quadriceps) and expressed as normalized absorbance units (AU).

### Statistical analysis

For WD studies, the independent factors were diet (NC vs. WD) and PA (SED vs. VWR). A diet by PA (2X2) analysis of variance (ANOVA) was employed as the global analysis for each dependent variable of interest. The comparison-wise error rate, *α*, was set at 0.05 for all statistical tests. When significant F ratios were found, a Fisher’s LSD post hoc analysis was used to distinguish differences among means. For MCK-PGC-1*α* mice compared to wild type (WT) littermates, Student’s *t-*test was used to determine differences among means. All data were analyzed using the Statistical Analysis System (SAS, version 9.3, Cary, NC), figures compiled using GraphPad Prism (La Jolla, CA), and data expressed as mean ± SEM.

## Results

### Demographic data for WD-induced obesity and voluntary activity

Phenotypic data are presented in Table2. Of note, WD animals consumed more calories than did NC-fed animals (1000 ± 60 kcal vs. 700 ± 30 kcal, respectively, across 8 weeks of intervention), but within SED and VWR groups there was no difference in caloric intake. Wheel activity was not different between NC-VWR and WD-VWR groups. WD-fed animals gained more body weight than did NC-fed animals regardless of PA (∼9 g total body weight difference); WD-fed groups showed greater epididymal fat mass than did NC-fed (∼76 mg/mm greater in WD groups). WD groups presented with larger gastrocnemius (0.6 mg/mm greater than NC), tibialis anterior (0.2 mg/mm greater than NC), and soleus (0.06 mg/mm greater than NC) muscles compared to NC groups.

**Table 2:** Descriptive variables for Western diet-induced obesity

Variable	NC-SED	NC-VWR	WD-SED	WD-VWR
Body weight (g)*	26.44 ± 0.60	26.25 ± 0.51	33.05 ± 0.61	36.06 ± 1.63
Epididymal Fat (mg/mm)*	18.5 ± 7.05	19.7 ± 7.05	86.0 ± 7.05	101.4 ± 7.05
Gastrocnemius (mg/mm)*	7.769 ± 0.201	7.788 ± 0.201	8.157 ± 0.201	8.453 ± 0.201
Tibialis Anterior (mg/mm)*	2.748 ± 0.070	2.502 ± 0.070	2.823 ± 0.070	2.723 ± 0.070
Soleus (mg/mm)*,†	0.517 ± 0.029	0.582 ± 0.029	0.557 ± 0.029	0.663 ± 0.029
Plantaris (mg/mm)	1.069 ± 0.032	1.129 ± 0.032	1.135 ± 0.032	1.157 ± 0.032
Extensor Digitorum Longus (mg/mm)	0.643 ± .0302	0.635 ± 0.030	0.678 ± 0.030	0.671 ± 0.030
Wheel activity (m/day)	N/A	525 ± 115	N/A	675 ± 275

All body weights and tissue weights reported as of time of tissue harvest. Tissue weights expressed as wet weight relative to tibia length (mg/mm).

* Main effect of diet.

† Main effect of VWR. Statistical markings placed with variable name.

### WD-induced obesity provokes glucose and exercise intolerance and increases markers of mitochondrial content

**Figure 1:**
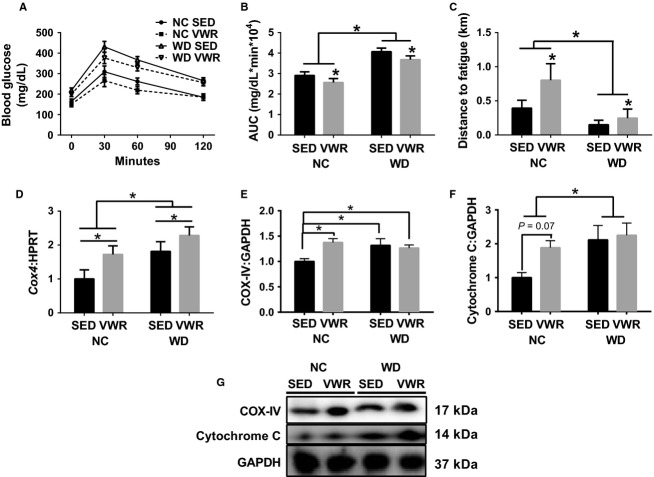
WD-induced obesity impairs glucose tolerance and results in greater mitochondrial content. (A) Response to glucose tolerance test. (B) Area under the curve (AUC) analysis from GTT. (C) Exercise tolerance measured by distance run to fatigue at end of study during a graded exercise test on treadmill. (D) mRNA content of *Cox4*. (E) Relative protein content of COX-IV. (F) Relative protein content of Cytochrome C. (G) Sample immunoblots corresponding to protein quantitation in E and F. All measured in gastrocnemius muscles and normalized to NC-SED. *P < 0.05 between indicated groups. Data are mean ± SEM. Representative immunoblot images for each protein of interest taken in order from same membrane.

Western diet-induced glucose intolerance, a surrogate of insulin resistance (main effect diet), and VWR groups showed a small but significant improvement in glucose tolerance compared to feed-matched SED animals (main effect VWR, ∼30–40 mg/dL × min × 10^4^, Fig.1A, B). WD impaired distance to fatigue during GXT compared to NC while VWR groups presented with longer distance to fatigue than SED groups (main effects of diet and VWR, 0.4 km longer in NC-VWR compared to NC-SED and 0.1 km longer in WD-VWR compared to WD-SED, Fig.1C). Concurrently, both mRNA and protein content of mitochondrial content marker COX-IV were greater in NC-VWR compared to NC-SED (72% and 38%, respectively). While COX-IV mRNA and protein were 80% and 32% greater in WD-SED compared to NC-SED (Fig.1D, E; mRNA equal main effects of VWR and diet). Cytochrome C protein content, though not significant, appeared to be greater in NC-VWR than NC-SED (*P* = 0.07), but was elevated above NC-SED by >200% in both WD groups (Fig.1F).

### WD feeding impairs induction of mitochondrial biogenesis markers

**Figure 2:**
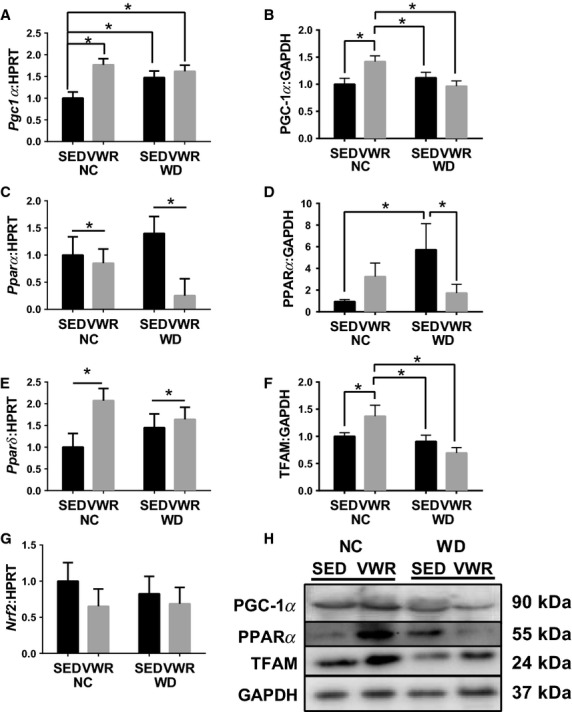
Content of mitochondrial biogenesis regulators in WD fed animals. (A and B) mRNA and protein content of PGC-1*α*. (C and D) mRNA and protein content of PPAR*α*. (E) mRNA content of PPAR*δ*. (F) Protein content of TFAM. (G) mRNA content of NRF2. (H) Sample immunoblots corresponding to quantitations in B,D,F. All measured in gastrocnemius muscles and normalized to NC-SED. *P < 0.05 between indicated groups. Data are mean ± SEM. Representative immunoblot images for each protein of interest taken in order from same membrane.

PGC-1*α* mRNA and protein content were significantly greater in NC-VWR compared to NC-SED (77% and 42%, respectively) and were not affected in WD-fed groups compared to NC-SED (Fig.2A, B). *Pparα* mRNA content demonstrated a main effect of VWR by which a reduction was seen in VWR groups compared to SED which appeared to be driven by WD groups (Fig.2C). PPAR*α* protein content was 5.7-fold greater in WD-SED animals compared to NC-SED and was no longer different from NC-SED with VWR (Fig.2D). *Pparδ* mRNA content exhibited a main effect of VWR where content was greater in VWR groups compared to feeding-matched SED groups which appeared to be driven by NC groups (Fig.2E). TFAM protein content was 37% greater in NC-VWR compared to NC-SED but was not different from NC-SED in either WD group (Fig.2F). *Nrf2* mRNA content was unaffected by diet or exercise (Fig.2G).

### Mitochondrial dynamics controllers are dysregulated in WD feeding

**Figure 3:**
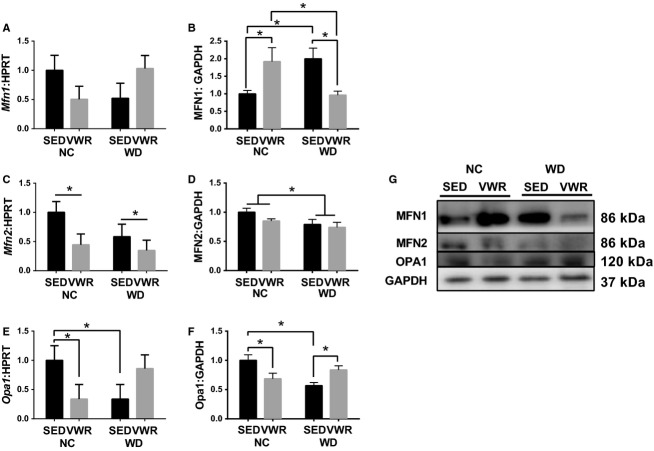
Content of mitochondrial fusion regulators in WD fed animals. (A and B) mRNA and protein content of MFN1. (C and D) mRNA and protein content of MFN2. (E and F) mRNA and protein content of Opa1. (G) Sample immunoblots corresponding to B,D,F. All measured in gastrocnemius muscles and normalized to NC-SED. *P < 0.05 between indicated groups. Data are mean ± SEM. Representative immunoblot images for each protein of interest taken in order from same membrane.

**Figure 4:**
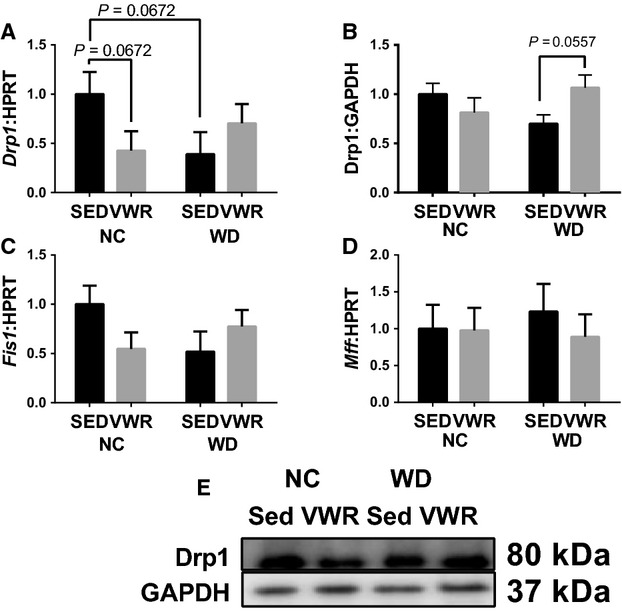
Content of mitochondrial fission regulators in WD fed animals. (A and B) mRNA and protein content of Drp1. (C) mRNA content of *Fis1*. (D) mRNA content of *Mff*. (E) Sample immunoblot corresponding to B. All measured in gastrocnemius muscles and normalized to NC-SED. *P < 0.05 between indicated groups. Data are mean ± SEM. Representative immunoblot images for each protein of interest taken in order from same membrane.

*Mfn1* mRNA content was not different among groups (Fig.3A). MFN1 protein content however was 92% greater in NC-VWR compared to NC-SED and appeared to be similarly greater in WD-SED (Fig.3B). *Mfn2* mRNA content exhibited a main effect of VWR where *Mfn2* was significantly lower in VWR groups compared to SED (Fig.3C). MFN2 protein content was approximately 20% lower in WD groups compared to NC-SED (main effect, Fig.3D). Opa1 mRNA and protein content demonstrated a similar pattern to one another. *Opa1* mRNA content was 33% of NC-SED in NC-VWR and was similarly down in WD-SED compared to NC-SED, WD-VWR was not significantly different from NC-SED (Fig.3E). Opa1 protein content was similarly lower in NC-VWR and WD-SED groups compared to NC-SED (∼35–40%) and WD-VWR was not different from NC-SED (Fig.3F). Regarding mitochondrial fission, *Drp1* mRNA appeared 60% lower in NC-VWR and WD-SED compared to NC-SED, though differences were not significant (*P* = 0.0672, Fig.4A). An interaction of diet and activity was seen for Drp1 protein content though differences between groups were not significant, however, WD-VWR appeared greater than WD-SED (*P* = 0.0557, Fig.4B). No significant change was seen in *Fis1* mRNA (Fig.4C), though the expression pattern resembled *Drp1*. No changes were seen in *Mff* mRNA content (Fig.4D).

### VWR promotes Beclin protein while WD feeding enhances autophagy flux

**Figure 5:**
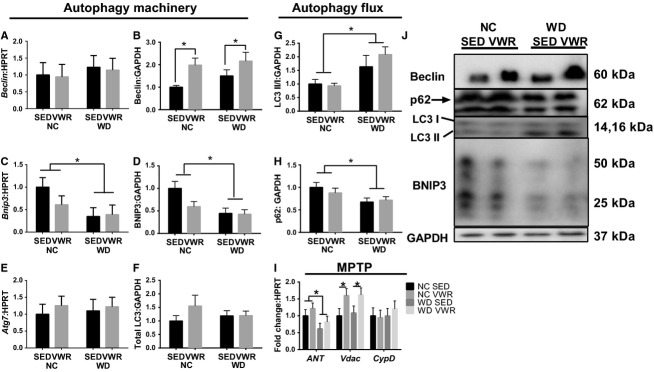
Autophagy machinery and flux markers in WD fed animals. (A and B) mRNA and protein content of Beclin. (C and D) mRNA and protein content of BNIP3. (E) mRNA content of *Atg7*. (F) Protein content of LC3. (G) LC3II:I ratio. (H) p62 protein content. (I) mRNA content of MPTP regulators. (J) Sample immunoblots corresponding to B,D,F,G,H. All measured in gastrocnemius muscles and normalized to NC-SED. *P < 0.05 between indicated groups. Data are mean ± SEM. Representative immunoblot images for each protein of interest taken in order from same membrane.

*Beclin* mRNA content was not affected by WD or VWR; however, Beclin protein content was significantly greater in VWR groups compared to SED (main effect, twofold and 50% in NC and WD feeding, respectively, Fig.5A,B). BNIP3 mRNA and protein contents were ∼50% lower in WD feeding groups (main effects of diet, Fig.5C,D). *Atg7* mRNA and total LC3 protein contents were not affected by WD or VWR (Fig.5E, F). Autophagy flux was enhanced by WD feeding as the LC3 II:I ratio was greater in WD (main effect diet, Fig.5G) and p62 protein content was less in WD groups compared to NC groups (main effect diet, Fig.5H). MPTP regulator *Ant* mRNA content was ∼40% lower in WD groups compared to NC (main effect diet, Fig.5I) while *Vdac* mRNA content was ∼60% greater in VWR groups compared to SED (main effect VWR, Fig.5I).

### PGC-1α promotes mitochondrial quality

**Figure 6:**
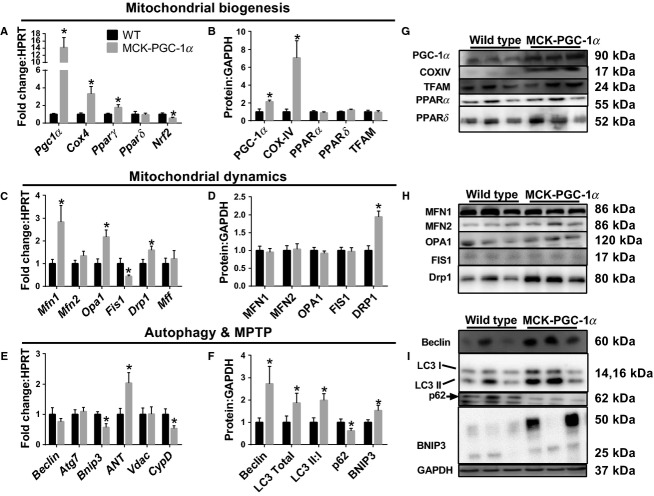
PGC-1*α* drives mitochondrial quality control mechanisms. (A and B) mRNA and protein content of mitochondrial biogenesis regulators in MCK-PGC-1*α* mice and wild-type (WT) littermates. (C and D) mRNA and protein content of mitochondrial dynamics regulators in MCK-PGC-1*α* mice and WT littermates. (E) mRNA content of autophagy and MPTP markers. (F) protein content of autophagy markers in MCK-PGC-1*α* mice and WT littermates. (G) Sample immunoblots corresponding to B. (H) Sample immunoblots corresponding to D. (I) Sample immunoblots corresponding to F. All measured in gastrocnemius muscles and normalized to NC-SED. *P < 0.05 between genotypes. Data are mean ± SEM. Representative immunoblot images for each protein of interest taken in order from same membrane.

As confirmation of genotype, MCK-PGC-1*α* mice presented with 10-fold greater *Pgc1α* mRNA and twofold greater PGC-1*α* protein than WT littermates (Fig.6A, B). COX-IV mRNA and protein contents were 3.3- and 7-fold greater, respectively, in MCK-PGC-1*α* mice than WT littermates (Fig.6A, B). mRNA content of *Pparδ* was not altered in MCK-PGC-1*α* mice while *Pparγ* (77% greater), *Nrf2* (50% lower), and *Tfam* (43% greater) were all significantly different in MCK-PGC-1*α* mice compared to WT littermates (Fig.6A). Protein contents of PPAR*α*, PPAR*δ*, and TFAM were not different between MCK-PGC-1*α* mice and WT littermates (Fig.6B). mRNA contents of *Mfn1* (2.8-fold greater), *Opa1* (twofold greater), *Fis1* (55% lower), and *Drp1* (60% greater) were all different in MCK-PGC-1*α* mice compared to WT littermates. *Mfn2* and *Mff* mRNA contents were not different between genotypes (Fig.6C). Protein contents of MFN1, MFN2, Opa1, and Fis1 were not different between genotypes (Fig.6D). Drp1 protein content was 200% greater in MCK-PGC-1*α* mice compared to WT littermates (Fig.6D). mRNA contents of autophagy-related genes *Atg7*, *Beclin*, and *Bnip3* were not different between genotypes (Fig.6E). Beclin and LC3 protein contents were 170% and 87% greater in MCK-PGC-1*α* mice than WT littermates, respectively, while LC3II:I ratio was twofold greater and p62 protein content was ∼50% lower in MCK-PGC-1*α* mice compared to WT littermates (Fig.6F). MPTP regulator *Ant* mRNA content was twofold greater and *CypD* 50% lower in MCK-PGC-1*α* mice compared to WT littermates (Fig.6G).

## Discussion

To our knowledge, this is the first study to report the comprehensive impacts of diet-induced obesity/insulin resistance on the cumulative mitochondrial quality control mechanisms (biogenesis, fusion, fission, autophagy, and MPTP). Additionally, we show that PGC-1*α*, the putative master regulator of mitochondrial biogenesis, is sufficient to promote multiple aspects of mitochondrial quality control. The current study provides critical new information detailing points of dysregulation in mitochondrial quality control in metabolic disease while providing evidence for potential mechanistic control of these systems by PGC-1*α*.

In this investigation, WD, designed to mimic human diets in Western societies, induced obesity and glucose intolerance (surrogate of insulin resistance). Moderate lifestyle PA (VWR, <1 km/day) partially ameliorated these effects on glucose intolerance but not obesity. That this small amount of activity provided some protection against glucose intolerance may be vital to utilization of PA to combat insulin resistance. Importantly, WD animals exhibit greater mitochondrial content than NC, (COX-IV and Cytochrome C). Despite elevated mitochondrial content, WD animals exhibit severely reduced treadmill performance (∼30–40% of distance run in corresponding NC groups), though VWR improved performance compared to feed-matched SED animals. Interestingly, WD-VWR animals performed similar wheel running activity as NC however exercise tolerance was severely blunted. This could be due to a combination of the moderate activity levels being superseded by the lipid overload of the diet and should be noted that wheel activity was voluntary. As such, following diet-induced obesity animals exhibit glucose and exercise intolerance despite increased mitochondrial content. As prior work has demonstrated reduced mitochondrial quality and function and increased mitochondrial ROS emission (Kelley et al. 2002; Anderson et al. 2009; Yokota et al. 2009; Laker et al. 2014) this now appears to occur despite elevated mitochondrial content. In contrast, NC animals exhibit VWR-induced increase in mitochondrial content concurrent to improvements in glucose and exercise tolerance. Therefore, we focused our investigation on the subsequent regulatory steps of mitochondrial quality control including those responsible for mitochondrial turnover (biogenesis and autophagy) and those required for maintenance of an interconnected network (fusion and fission) to determine faults in the maintenance of mitochondrial quality and health.

### WD-induced obesity impairs regulation of mitochondrial quality control

First, PGC-1*α* was significantly induced by 4 weeks of PA in NC animals corresponding to changes in mitochondrial content. However, exercise induction of PGC-1*α* in WD was abolished, and mirrored by TFAM protein. While PPAR*α* protein content is elevated by WD, this is likely a response to the lipid overload of the diet, as the system attempts to handle the excess lipid. Interestingly, recent data suggest regulation of mitochondrial dynamics via PPAR*α* agonism (Zolezzi et al. 2013) as well as its primary function in regulating lipid handling genes. VWR inductions of PPAR*δ* and TFAM are similar to our prior findings in exercise training in humans and obese rats, interestingly here we see impaired induction of TFAM in the WD-induced obese animals which was not seen in our work in obese Zucker rats (Greene et al. 2012, 2014). These results demonstrate that WD feeding promotes an increase in mitochondrial content but impairs functional regulation of mitochondrial biogenesis suggesting that elevated mitochondrial content is likely the result of downstream effects from biogenesis. Similarly, we previously observed increased mitochondrial content in the obese Zucker rat (Nilsson et al. 2010; Greene et al. 2014). In that work we concurrently measured the fractional synthesis rate of mitochondrial protein and observed resistance to exercise-induced mitochondrial anabolism in obese animals. Together our evidences now suggest that increases in mitochondrial content in skeletal muscle of obese animals occur in both genetic and diet-induced models and despite impaired induction of key mitochondrial biogenesis regulators. It is important to note, however, that we have not directly measured mitochondrial biogenesis itself in the current study and can therefore only speak to limitations in the regulation of biogenesis.

Second, regulation of mitochondrial fusion appears dysfunctional in WD feeding. Most importantly, protein expressions of MFN2 and Opa1 are reduced in WD suggesting an impaired ability to promote mitochondrial fusion. By contrast, VWR-induced enhanced expression of MFN1 protein in NC animals likely suggesting an attempt to upregulate capacity for mitofusion. Interestingly, we see few changes in the expression of fission factors. Therefore, we cumulatively see reduced capacity to perform fusion with no significant alterations in fission. Therefore, the balance seems to shift to favor mitochondrial fission (less fusion relatively) and thus degeneration of the mitochondrial network. Again, we are restricted to interpreting the key regulators of these processes. Direct assessments of mitochondrial dynamics require time-lapse microscopy and have been met with limited success in adult skeletal muscles with few known publications reporting to have accomplished this feat (Luo et al. 2013). However, future investigations should strive to define the balance of mitochondrial dynamics by such techniques to determine if fission is indeed favored functionally as well as by the alterations in regulators.

Third, autophagy is aberrant in WD feeding. We observed no significant alterations in the mRNA expression of the autophagy genes *Beclin* and *Atg7* potentially suggesting that in skeletal muscle alterations in general autophagy are not mediated by transcriptional control but through translational and flux-based controls. To that point, we note, similar to Lira et al. (Lira et al. 2013), that Beclin protein is promoted by voluntary activity, regardless of diet, which may indicate a PA-induced improvement in the ability to induce autophagy. Additionally, WD feeding enhanced total autophagy flux seen in canonical flux markers of increased LC3 II:I ratio and reduced p62 content. Unlike the prior report by Lira et al. (Lira et al. 2013), we did not observe a promotion of autophagy flux by PA. This difference is likely due to relatively moderate amounts of activity performed by animals in our study which were considerably less than in that prior investigation. Interestingly, total autophagy flux is elevated in WD. Based on impaired expression of biogenesis regulators and increase in mitochondrial content, we expected impaired autophagy flux following WD. Impaired flux would suggest compromised ability to remove damaged mitochondria. Thus, increased mitochondrial content would be the result of the accumulation of damaged and degenerated mitochondria which have previously been observed to occur in lipid overload models (Laker et al. 2014). However, these are markers of total autophagy and are not specific to mitophagy. Therefore, elevated total autophagy flux may not be indicative of autophagic removal of mitochondria. To that point we observe reduced BNIP3 in WD animals. Impaired BNIP3 contents in WD animals suggest that even though total autophagy flux is elevated in WD animals, they are likely unable to efficiently remove damaged mitochondria. As these interpretations are based on a single target, future investigation is necessary to better understand the direct effects of WD feeding on specific mitophagy. We suggest an important new step in these investigations is to directly measure the mitophagy flux, however such measurements require, at the least, colocalization of the mitochondria and autophagosome but this would merely indicate the targeting of mitochondria for autophagy and not the resolution of that autophagy.

Finally, we investigated transcriptional control of MPTP regulators. While *CypD* is not affected by VWR or WD we do demonstrate reduced mRNA expression of *Ant* in WD. This may indicate reduced capacity for opening of the MPTP by WD, which may contribute to degeneration of the mitochondrial network. Importantly, *Vdac* mRNA expression is promoted by VWR regardless of diet which may suggest that PA promotes some function of MPTP capacity.

**Figure 7:**
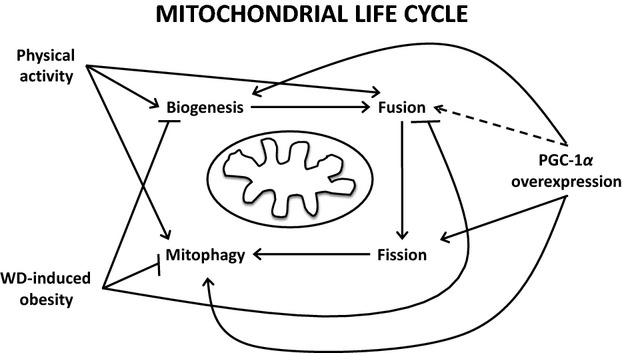
Mitochondrial quality control and the impacts of WD-induced obesity, VWR, and PGC-1*α* overexpression on these processes. All three conditions were associated with increased total mitochondrial content, however, impacts on mitochondrial biogenesis, fusion, fission, and mitophagy appear to differ between conditions. Key: Arrow = promotes process; Crossed line = inhibits process; Dashed line = transcriptional evidence only.

Combined, WD-induced obesity impairs glucose and exercise tolerance concurrent to increased mitochondrial content. These impairments are associated with dysfunctions in the regulation of mitochondrial biogenesis, dynamics, autophagy, and MPTP. While our focus was to examine regulation of the mitochondrial quality control mechanisms, previous reports have detailed impaired mitochondrial quality (Laker et al. 2014), function (Yokota et al. 2009), and ROS emission (Anderson et al. 2009) which may be able to be corrected by exercise (Konopka et al. 2015). Our data add to those findings by detailing how diet-induced obesity impacts mitochondrial quality control regulatory factors. In fact, based on these data, we suggest obesity-induced increase in mitochondrial content is likely an accumulation of damaged and fissed mitochondria unable to be cleared by mitophagy. In contrast, moderate PA in healthy animals enhances mitochondrial content concurrent to promotion of canonical biogenesis regulators and at least portions of mitofusion and autophagy machineries suggesting PA-induced enhancements in mitochondrial network maintenance. Therefore, PA appears to enhance mitochondrial content through biogenesis and promoting health of the network through enhanced fusion and clearance, while WD-induced obesity enhances mitochondrial content via reduced induction of biogenesis and fusion with impaired mitophagy (Fig.7). As our studies focused on signaling regulation future experiments should attempt to directly tie these dysregulations to direct measures of mitochondrial quality and function. It stands to reason that targeting of mitochondrial quality rather than content may provide an excellent opportunity to repair glucose and exercise tolerance in lipid overload conditions such as WD. Future studies should examine the interactive impacts of mitochondrial quality control and canonical insulin signaling, specifically the inhibitory tyrosine phosphorylation of the IRS. Therefore, we next sought to examine the mechanisms by which mitochondrial quality control regulators are altered. As PGC-1*α* is the master regulator of mitochondrial biogenesis and recent reports suggest a role of PGC-1*α* in dynamics (Cartoni et al. 2005) and autophagy (Lira et al. 2013), we hypothesized that PGC-1*α* is sufficient to promote the total mitochondrial quality control.

### PGC-1α is sufficient to promote multiple aspects of mitochondrial quality control

First, we confirmed the robust expression of both PGC-1*α* and COX-IV at gene and protein levels in MCK-PGC-1*α* mice. However, protein expression of downstream regulators of mitochondrial biogenesis, TFAM, and PPAR*α* is unaffected in this model, while gene expression of *Tfam* and *Pparγ* was significantly upregulated. These data suggest that PGC-1*α* overexpression promotes transcription of some downstream regulators of biogenesis but this is not necessarily leading to a measurable change in protein. Second, Cartoni et al. (Cartoni et al. 2005) demonstrated transcriptional induction of *Mfn1* and *Mfn2* following exercise which appeared to be regulated by PGC-1*α* via ERR*α*. At the transcriptional level, we similarly observed the induction of *Mfn1* and *Opa1* with a nonsignificant increase in *Mfn2* in MCK-PGC-1*α* mice. However, induction of mitochondrial fusion components was not seen at the protein level. Therefore, PGC-1*α* indeed promotes transcriptional regulation of mitochondrial fusion as observed (Cartoni et al. 2005); however, this does not translate to measurable changes in mitofusion proteins. Third, we noted that mitofission factor Drp1 mRNA and protein were elevated in MCK-PGC-1*α* mice. Therefore, under sedentary cage control conditions, PGC-1*α* transcriptionally promotes mitofusion and fission, however, the translation of these gene products to protein is repressed by an as yet unidentified posttranscriptional mechanism. Fourth, we observed no significant alterations in transcript levels of autophagy-related genes. However, unlike previous findings (Lira et al. 2013) we see an increased Beclin protein in MCK-PGC-1*α* mice. Similar to that report, we show enhanced autophagy flux (increased LC3 II:I ratio and reduced p62 protein), and enhanced mitophagy (BNIP3), in PGC-1*α* overexpressing mice. These findings suggest a lesser role for PGC-1*α* in transcriptionally mediating autophagy machinery but confirm evidence of PGC-1*α*-mediated enhanced autophagic flux. Finally, MPTP factors displayed alternate responses to PGC-1*α* overexpression. *CypD* was downregulated while *Ant* was twofold greater in MCK-PGC-1*α* compared to WT. We are uncertain as to the difference in this regulation; however, *CypD* appears to be the most critical of these factors for the MPTP, and therefore a reduction in *CypD* would likely result in a reduced susceptibility to MPTP. We should acknowledge that at this time we are unable to fully discern if these effects of PGC-1*α* overexpression are direct effects resultant of PGC-1*α* itself or mediated by increased mitochondrial content. Interestingly, all three conditions examined here; VWR, WD-induced obesity, and PGC-1*α* overexpression lead to increased mitochondrial content. However, the means by which each condition impacts total mitochondrial quality control and the mitochondrial life cycle is not uniform (Fig.7).

Our findings of PGC-1*α*’s role in regulating mitochondrial quality control confirm prior evidences for PGC-1*α* in regulating mitochondrial biogenesis and autophagy. Interestingly, our data suggest a role of PGC-1*α* in transcriptionally regulating mitochondrial dynamics and MPTP. Our conundrum, however, is that the transcriptional regulation of mitochondrial fusion by PGC-1*α* did not translate to elevated protein. Additional investigation into the mechanism by which translation of these mRNAs is inhibited will prove vital to efforts to promote mitofusion. Our data are in NC-fed sedentary animals with no additional intervention. Previous reports demonstrate that muscle-specific overexpression of PGC-1*α* in sedentary conditions exacerbates diet-induced insulin resistance (Choi et al. 2008; Summermatter et al. 2013). We speculate the lack of translation of mitochondrial fusion proteins may be functionally due to the overabundance of mitochondria relative to the animal’s living condition which does not dictate the presence of this high volume of mitochondria. Therefore, in these conditions maintaining a large interconnected mitochondrial network is unnecessary, as such, we see a shift in the balance in the protein levels of mitochondrial dynamics regulators toward fission. It would, therefore, be of great interest to determine if PGC-1*α* overexpression promotes mitochondrial fusion at the protein level in physically active animals that do have a greater bioenergetic need placed upon the mitochondria. This conundrum deserves a more specific examination to determine how PGC-1*α* promotion of mitochondrial quality is likely lost with inactivity. Considering the combined transcriptional and translational evidences, we suggest that PGC-1*α* promotes multiple aspects of mitochondrial quality but this promotion is compromised in cases of lipid overload or inactivity. Future experiments should directly assess the role of PGC-1*α* to promote these factors in a WD and PA model. These studies provide a strong basis for PGC-1*α* control over mitochondrial quality regulators. We should note that PGC-1*α* family transcription factors do exhibit predicted binding to the promoter regions of multiple genes throughout these processes (DECODE Database, SA Biosciences) and therefore PGC-1*α* mediated transcriptional promotion deserves significant future examination.

In summary, the current study is the first to comprehensively examine mitochondrial quality control regulators throughout biogenesis, fusion/fission dynamics, autophagy, and MPTP in diet-induced obesity. We demonstrate derangements in the expression of controllers involved in each of these processes in WD-induced obesity. These derangements suggest impaired mitochondrial biogenesis, despite enhanced mitochondrial content, with a shift in mitochondrial dynamics to favor fission over fusion and impaired mitophagy. We further see that only moderate voluntary activity provided a small protection against glucose and exercise intolerance and was able to promote expression of some of these factors in WD. Finally, PGC-1*α* may not only control biogenesis but is sufficient to promote transcription of regulators of mitochondrial fusion and fission and basal autophagy flux. As prior studies have detailed impairments in mitochondrial quality and function, these data provide an important new step to discerning the means by which diet-induced mitochondrial degeneration may be targeted to ameliorate obesity and insulin resistance. Our data indicate the importance of looking beyond mitochondrial content and biogenesis in the development of metabolic disease to focus on promoting mitochondrial quality.
